# Space Radiation Research Unit, International Open Laboratory in NIRS

**DOI:** 10.1093/jrr/rrt204

**Published:** 2014-03

**Authors:** Yukio Uchihori, Tom K. Hei, Teruaki Konishi, Alisa Kobayashi, Hisashi Kitamura, Satoshi Kodaira, Shingo Kobayashi

**Affiliations:** National Institute of Radiological Sciences, Chiba 263-8555, Japan

**Keywords:** space radiation, dosimetry, HIMAC, microbeam

## Abstract

The radiation environment encountered by astronauts during spaceflight is far more complex than any radiation field existed on Earth. Space crew living and working in the International Space Station (ISS) are exposed to a mixed radiation field comprises primary high-energy cosmic rays, including energetic protons and heavy ions, and to secondary radiations, including energetic neutrons, produced when the primary radiation interacts with the mass of the space station and its contents. The doses of ionizing radiation received by astronauts and cosmonauts aboard the ISS are many times greater than those received by radiation workers on the ground. Exposure to ionizing radiation in space includes high LET events than can produce significant biological damage in human cells and tissues, and thus represents an important risk to space crew health and safety.

The Space Radiation Research Unit at the National Institute of Radiological Sciences (NIRS) includes both physicists and radiation biologists and there is extensive collaboration between these two groups. This provides us with the expertise needed to investigate the effects of space crew exposure to the highly complex, mixed radiation environment encountered in space. In addition, NIRS is home to a heavy ion accelerator, HIMAC and the Medical Cyclotron that can be used to simulate various components of the space radiation environment. Recently, we have developed a medium energy proton radiation field using the NIRS Medical Cyclotron. [How about a sentence or two on the significance of this proton facilities.] In addition, NIRS has also developed a high precision tool, the Single Particle Irradiation System to Cell (SPICE) microbeam facility, for use in investigating various radiobiological endpoints, including the bystander effect and the adaptive response of various cell types, *C*aenorhabditis *elegans* and in Medaka fish. Some of these research activities are described in these proceedings [1, 2].

The Space Radiation Research Unit at NIRS has extensive international collaborations with researchers in China, Germany, Russia, USA, Sweden, Austria and other countries. In particular, collaborations with our Russian colleagues has led to opportunities to expose our dosimeters aboard the ISS and to numerous other experiments in the frame of the IOL, as described more fully elsewhere in these proceedings. We believe that the framework of the IOL is very powerful and productive and we plan to continue our collaborative research with our international colleagues.

## Figures and Tables

**Figure RRT204F1:**
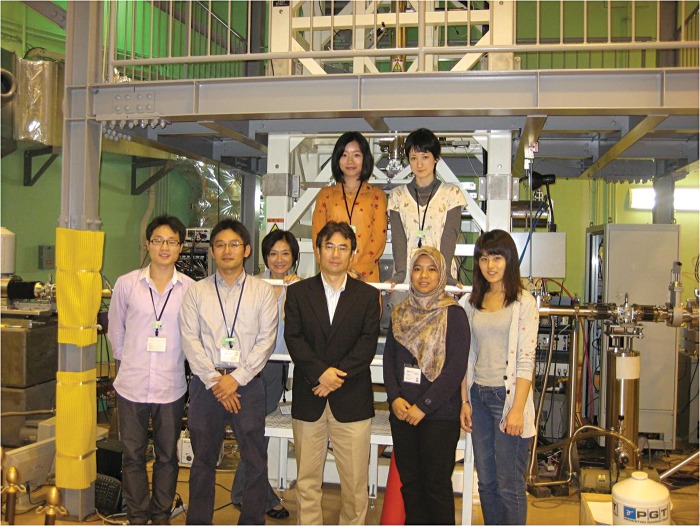
International collaborators from Fudan University in China, the City University of Hong Kong and from Malaysia. With Drs. Yukio Uchihori and Teruaki Konishi

**Figure RRT204F2:**
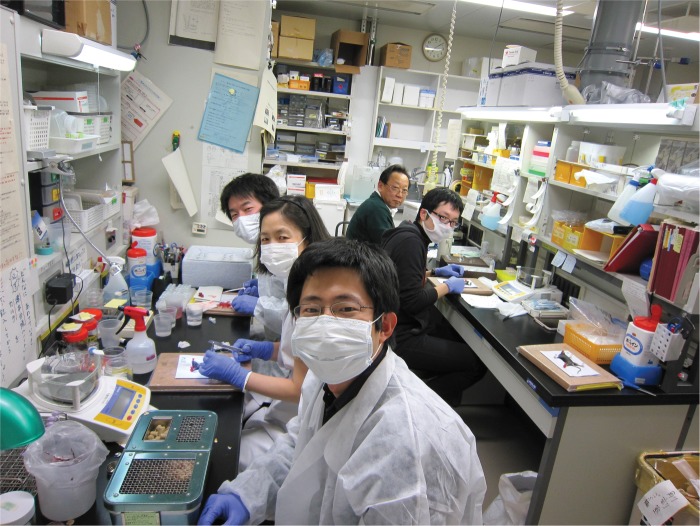
Visiting doctoral student Yufei Chai from Professor Tom Hei's laboratory at Columbia University Medical Center conducting studies with NIRS investigators.

**Figure RRT204F3:**
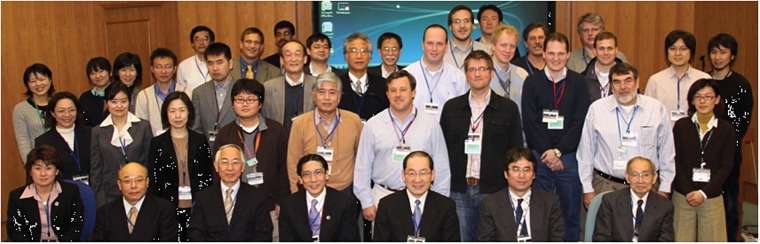
The inaugurial workshop of the Space Radiation Unit of the International Open Laboratory March 2009.

## References

[1] Konishi T, Oikawa M, Suya N *J Radiat Res*, this proceeding..

[2] Yang G, Konishi T, Kobayashi A *J Radiat Res*, this proceeding..

